# Multimodal mobile brain and body imaging for quantification of dance motor sequence learning

**DOI:** 10.1016/j.mex.2025.103324

**Published:** 2025-04-19

**Authors:** Russell W. Chan, Victoria Lakomski, Johannes V.R. Pannermayr, Emma Wiechmann, Jan-Willem J.R. van ‘t Klooster, Willem B. Verwey

**Affiliations:** a Faculty of Behavioural, Management and Social Sciences, Section Cognition, Data and Education, University of Twente, Enschede, Netherlands; b Division of Clinical Geriatrics, Department of Neurobiology, Care Sciences and Society, Karolinska Institutet, Huddinge, Sweden

**Keywords:** Motor neuroscience, Motor sequence learning, 3D modelling, Discrete sequence performance task, Centre of mass, EEG, MoBI, Kinematics, Multimodal mobile brain and body imaging for the quantification of dance motor sequence learning

## Abstract

Understanding motor learning in naturalistic settings presents a key challenge in neuroscience. While paradigms like the Discrete Sequence Production (DSP) task have advanced our knowledge, investigating more naturalistic tasks like dance with multi-limbed coordination can help further advance the understanding of complex mechanisms. It can advance motor learning by providing more profound insights into coordination dynamics, movement execution, balance, and decision-making. We have developed a modified DSP methodology that replaces keyboard pressing with dance-stepping, allowing simultaneous electroencephalography (EEG), behavioral, and kinematic recordings to quantify neurophysiological and motor dynamics. Using an E-Prime^Ⓡ^ script in a go/no-go approach, our method accommodates both a setup with minimal hardware and also a scalable approach with markerless motion capture and mobile EEG for neuroimaging. By leveraging Mobile Brain and Body Imaging (MOBI), we enhance the investigation of neuro-mechanisms underlying motor learning. We also discuss future directions and accessibility, including a publicly available video of the experimental procedure (https://youtu.be/zFP1rWJ2FJ8?si=DJ8q7fbfhltSLehz), enabling broader replication and application of our methodology.

• Conversion of the key-press Discrete Sequence Production task to a dance version, as an applied way to investigate motor sequence learning
• Multimodal investigation with motion capture and electroencephalography for kinematics and neuroimaging
• Full scripts in E-Prime^Ⓡ^ are freely downloadable and video link showcases experiment conduct

## Graphical abstract

**Figure:**
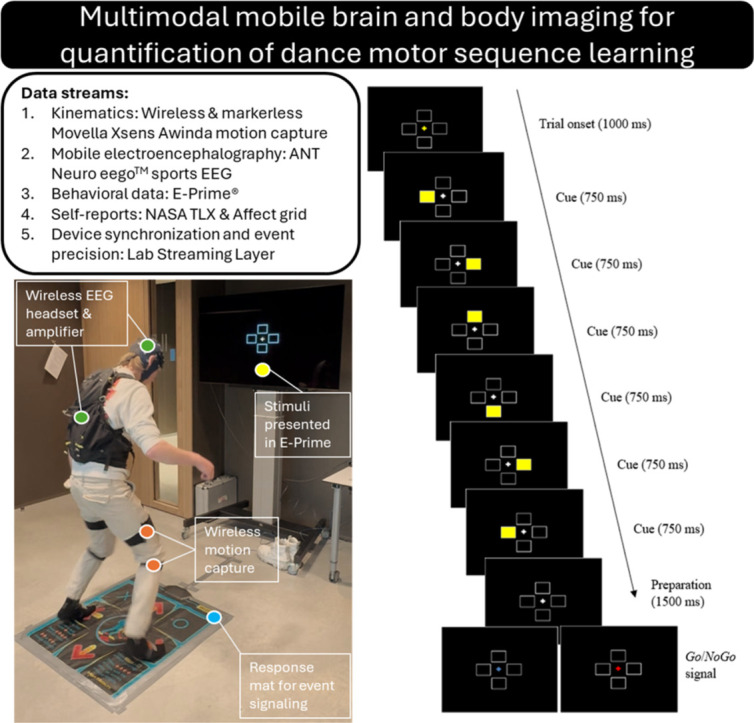

Specifications table

**Table:** 

Subject area:	Neuroscience
More specific subject area:	Motor neuroscience
Name of your method:	Multimodal mobile brain and body imaging for the quantification of dance motor sequence learning
Name and reference of original method:	Verwey, W. B., [1]. Evidence for a multistage model of practice in a sequential movement task. Journal of Experimental Psychology: Human Perception and Performance, 25(6), 1693–1708. https://doi.org/10.1037/0096–1523.25.6.1693
Resource availability:	**Dance Discrete Sequence Production (DSP) task paradigm:** Procedural video: https://www.youtube.com/watch?v=zFP1rWJ2FJ8 E-Prime^Ⓡ^ Software: https://pstnet.com/product_category/experiment-design/ Experimental E-Prime^Ⓡ^ 2.0 script: https://www.osf.io/9x2te/files/osfstorage/62756dd9c6224026601bef15 Experimental E-Prime^Ⓡ^ 3.0 script: https://www.osf.io/9x2te/files/osfstorage/67c6e175037c9a570d08bf21 Dance mat USB input device: https://dancepadmania.com/deluxe/ JoyToKey software: https://joytokey.net/en/ **Mobile Electroencephalography:** ANT Neuro eego™ sports EEG: https://www.ant-neuro.com/products/eego-sports **Motion Capture Kinematics:** Movella Xsens MVN Awinda: https://www.movella.com/products/motion-capture/xsens-mvn-awinda **Device synchronization:** Lab streaming layer: https://labstreaminglayer.org/#/

## Background

One of the backbones of motor sequence learning experimentation is the Discrete Sequence Production (DSP) task [1,2]. This task uses a keyboard for learners to practice two short key pressing sequences, typically 3–7 stimuli, separated by breaks [3]. Small squares on a monitor represent keys, and learners press the corresponding key when a placeholder lights up. The DSP task involves: an extended practice phase for overlearning; counterbalanced sequences for participants to eliminate finger-specific effects; and sequences up to 7 key presses, allowing for preparation and development of integrated sequence representations [[4], [5], [6]].

Although key-press tasks are easy and insightful for sequence control processes with a plethora of theoretical frameworks [[7], [8], [9], [10], [11]], a broader challenge today is to develop more naturalistic paradigms. Everyday activities and sports require coordination of multiple body parts, which current motor sequence learning frameworks—derived mainly from keyboard tasks—don't fully explain. Heuer [12] emphasized that task constraints drive motor optimization, meaning outcomes vary with different limb modalities. Movements learned in one modality also may not transfer to others [13]. Designing experiments that account for multi-limb coordination is essential, as existing frameworks provide a foundation but need extension to encompass new cognitive and motor discoveries.

Du and Clark [14] adapted the Serial Reaction Time (SRT) task [15] into a foot-step paradigm, quantifying pre-movement center of mass anticipatory shifts as an indicator of explicit sequence knowledge. Building on this foundation, Olivier et al. [16], assessed the feasibility of the footstep-based SRT design across diverse populations, demonstrating its applicability beyond the keyboard. In a tennis sporting context, O'Connor et al. [17], embedded the SRT sequence in a serve return task, although they did not apply any kinematics or neurophysiological measures to understand mechanisms. Recognizing this need for more ecologically valid approaches for motor sequence learning research, we propose the development of a multimodal Mobile Brain/Body Imaging (MoBI) framework that integrates motion capture kinematics and neuroimaging.

Beyond measuring behavioral response times, modern markerless motion capture systems (e.g., Movella Xsens) enable more in-depth analysis of time-space factors influencing multi-limbed coordination and body movements essential for sequence representation. This kind of data could help explain individual differences and reveal constraints within the DSP task [12]. In the keyboard DSP task, sequence learning characteristics are derived from limited degrees of freedom (DoF) to unveil underlying sequencing properties. When the body is used to “dance” in the Dance-DSP task we designed, lower limb effectors are coupled with an uneven number of response locations, which invokes dynamically changing DoFs to optimize sequence execution. Different participants may use varying limbs or movements (e.g., jumps/rotations) for the same stimuli, which key-press tasks cannot reveal. Coupled with neuroimaging technology (e.g., ANT Neuro's eego^TM^), this approach captures neurophysiological data such as electroencephalography (EEG) or functional near-infrared spectroscopy (fNIRS) to further understand mechanisms in motor learning.

With these motivations, we developed a new DSP task approach, resembling a dance, using advanced techniques to capture and quantify behavioural, kinematic, and neuroimaging data. Our MoBI approach improves the ecological validity of motor sequence learning research and opens novel possibilities for enhancing theoretical foundations and applied areas like manufacturing and rehabilitation.

## Method details

### Overview of experiment and synchronization of device protocols using Lab Streaming Layer

**Fig. 1:**
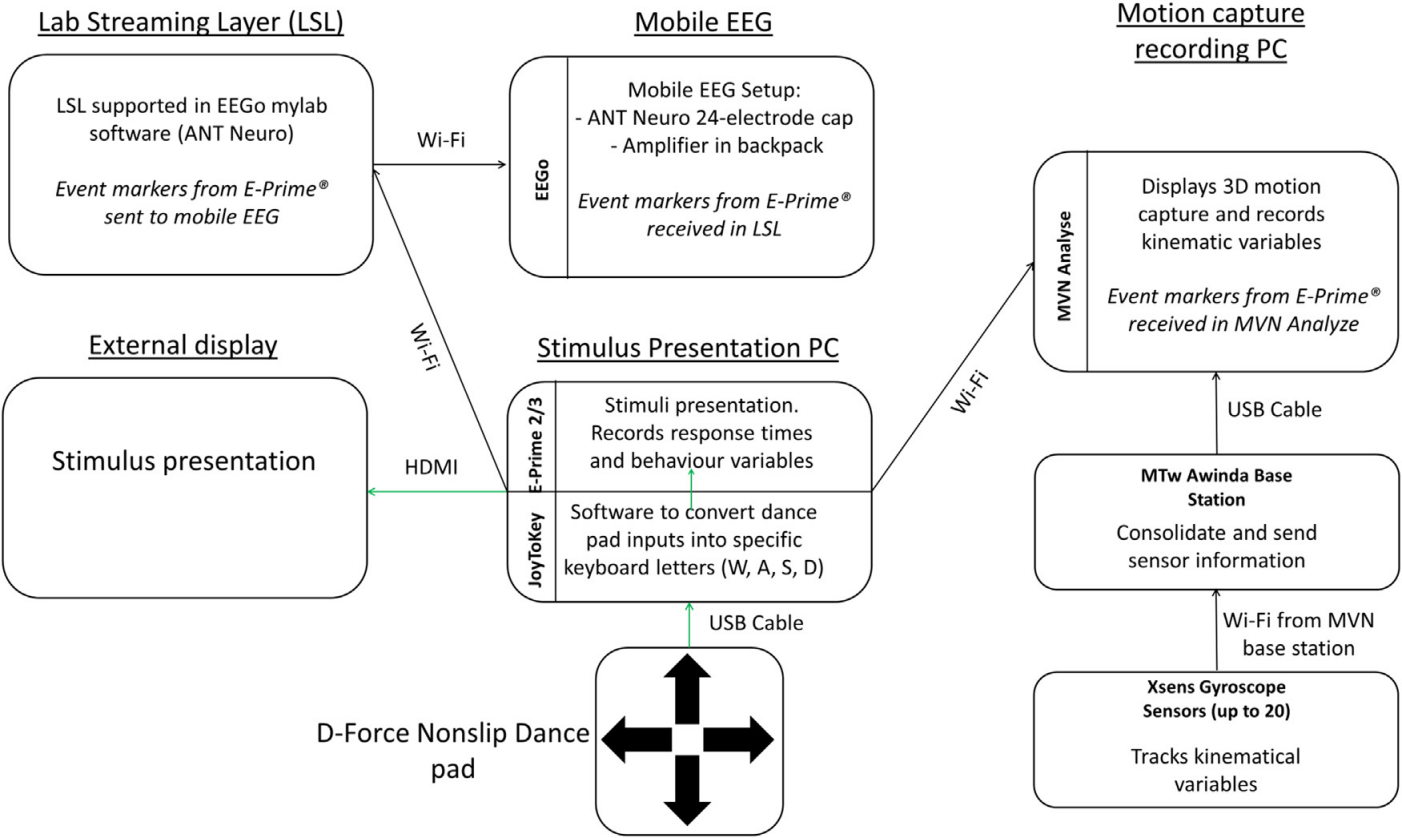
Overview of equipment and respective connections. The basic setup (in green arrows) only requires a stimulus presentation PC to run E-Prime^Ⓡ^ and record behavioral variables, including step-level accuracy and response times of participants. The bottom-right side of the diagram shows that Movella Xsens motion capture sensors that can be configured with up to 20 sensors in a full suit for recording biomechanical kinematics such as limb segment, sensor vector displacement, velocity, and acceleration. The sensors communicate through Wi-Fi with the MTw Awinda base station, which connects to the MVN Analyze recording software via a USB-A cable. Importantly, E-Prime^Ⓡ^ sends event markers through line code via a Wi-Fi to MVN Analyze for indexing different movement moments (e.g. each step). In the top left and center of the diagram, EEG can be connected to the setup for neuroimaging. Lab streaming layer is required in our setup for E-Prime^Ⓡ^ to send event markers to the ANT Neuro mylab EEG recording software. In this way, all stimuli and response events are synchronized and recorded in EEG and motion capture data.

As an overview, we outline the complexities of combining stimulus presentation, EEG and motion capture that requires synchronization via the open-sourced Lab Streaming Layer software (LSL) for event precision [18]. A critical aspect of the current method is to know when participants are responding for each step. A specific Lab Streaming Layer (LSL) code (in the E-Prime^Ⓡ^ 3.0 script) was written to send markers from E-Prime^Ⓡ^ to the EEG recording. The eego^TM^ mylab software supports LSL by default and the markers were sent via a local Wi-Fi network – Netgear Nighthawk AX5400 Wi-Fi 6 Router not connected to the internet from E-Prime^Ⓡ^. This same local Wi-Fi also transmitted event markers that were sent from E-Prime^Ⓡ^ to MVN Analyze software so that the kinematics data was marked synchronously alongside the EEG. Fig. 1. below shows a schematic overview of how all the equipment are connected and synchronized with LSL.

### Dance Discrete Sequence Production task in E-Prime^Ⓡ^

**Fig. 2:**
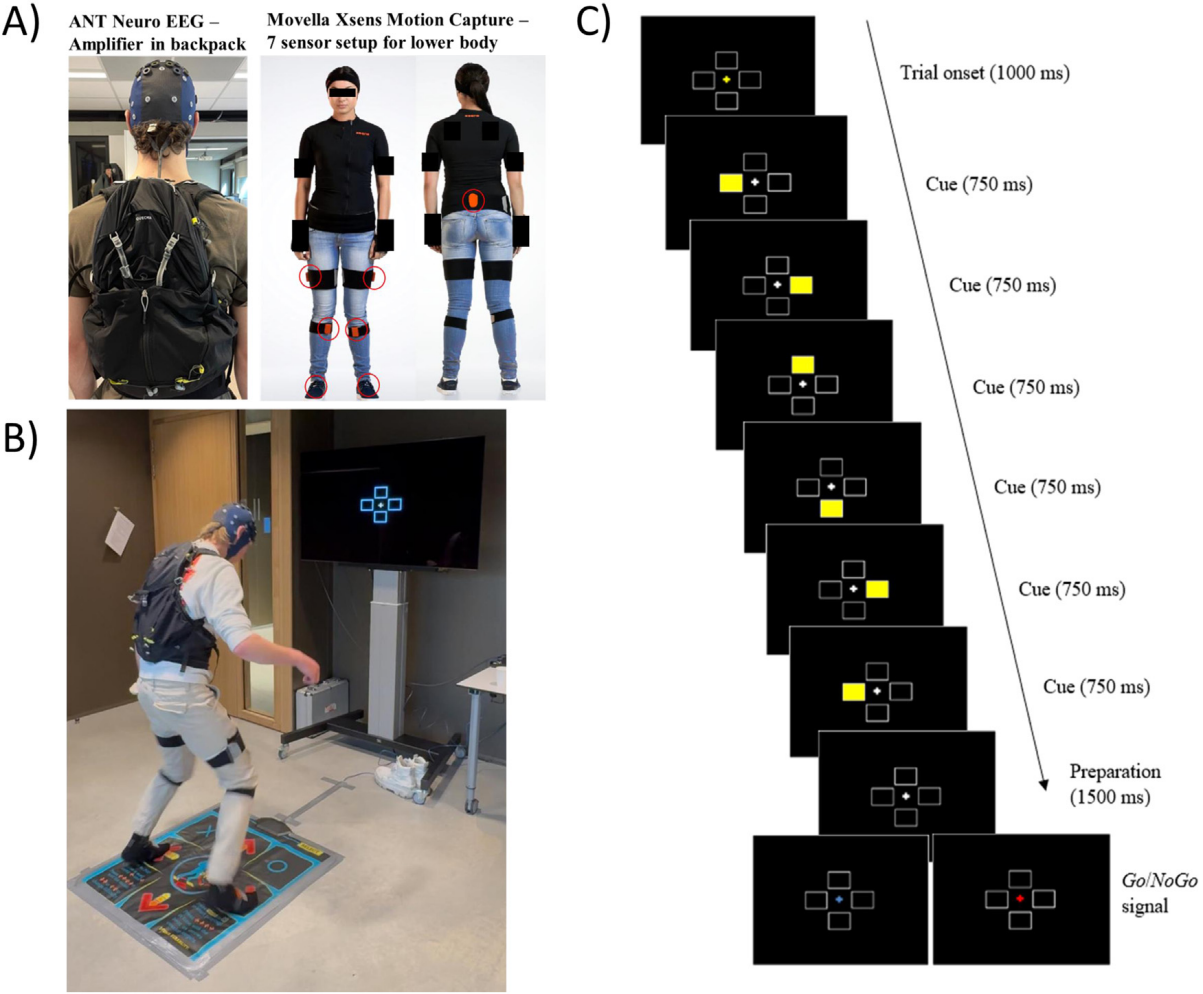
Experimental set-up and stimuli. (A) Our Mobile Brain/Body Imaging system consisted of ANT Neuro's eego^TM^ sports EEG, secured in a commercial runner’s backpack and Movella’s Xsens MTw Awinda. (B) During the dance DSP task, participants stand on a mat ∼120 cm away with an overall viewing angle of the stimulus area of ∼30° After the stimuli presentation, participants reproduce the sequence by stepping on the spatially corresponding areas on the dance mat. (C) An example of the Dance Discrete Sequence Production task sequence that is presented from the onset of stimuli to the Go/NoGo signal. The duration of presentation is indicated for each phase.

We used E-Prime^Ⓡ^ as our stimulus presentation software due to its timing consistency in collecting reliable behavioral data – Response Time and Accuracy, and relative ease in programming. The full script was programmed in E-Prime^Ⓡ^ Version 2.0.10.356. The .es2 file – (https://osf.io/9x2te/files/osfstorage/62756dd9c6224026601bef15), es.3 file – (https://osf.io/9x2te/files/osfstorage/67c6e175037c9a570d08bf21), and curated on Github (https://github.com/Eggcite/Dance_DSP) for adaptation and replication. The main difference between the usual key-press DSP and the current Dance-DSP task is the stimulus and response layout. The usual horizontal row of boxes now spatially corresponds to the dance mat design (↑, ↓, → and ←) with a center neutral position (see Fig. 2C). We used a high-quality commercially available dance mat (Nonslip Dance Pad Version 5 from d-Force) (See Appendix 1 for full equipment list). Participants start the Dance-DSP task standing with both feet on the center area of the dance mat whilst stimuli are presented on a wide screen television – LG model nr. OLED77CX6LA with a diagonal size of 77 inches (1.96 m), 3840 × 2160 pixel resolution in HDR color, with a screen refresh setting of 120 Hz. One can use any screen, so long as the participant has approximately 1 – 1.5 m from the screen for adequate space. In our case, participants stand approximately 1.20 m from the display to the center of the dance mat. The viewing angle of the display is approximately 120° and the visual angle dimension of each box is 2° × 2° with an overall viewing of the stimulus area approximately 30° The TV screen and the dance mat are connected to a Windows laptop that executes the Dance-DSP task script (see Fig. 1 for connectivity of all devices). The setup uses a freeware application called JoyToKey (https://joytokey.net/en/), which converts input from the responses to assigned keys for E-Prime^Ⓡ^ to register on the dance mat. Thereafter, E-Prime^Ⓡ^ recognizes the directional keys as input from a traditional keyboard. We mapped the ↑, ↓, → and ← as W, S, D, A respectively as input for responses.

Each trial consists of six stimuli (can be reduced/ increased) that are presented by the successive lighting up of the rectangular placeholders on the screen. As shown in Fig. 2C, first the default screen is presented with a cross in the middle lighting up in yellow for 1000 milliseconds (ms) upon which six rectangles take turns lighting up in yellow for 750 ms each (cf., [19]). Next, participants see the default screen for another 1500 ms after which the cross in the middle lights up in either blue (Go) or red (NoGo). In the case of a Go stimulus, participants reproduce the sequence they just saw by taking six steps on spatially corresponding areas on the dance mat. In the case of a NoGo stimulus, the waiting time lasts three seconds until the next sequence is displayed. As outlined in De Kleine and Van der Lubbe [19], the break during which participants wait for a signal makes it possible to separate sequence preparation and execution. The frequency of Go or NoGo stimuli was 92 % and 8 %, respectively. If the participant moved before the Go signal, a “Too early!” message is displayed to halt the current trial, and then the next trial is shown. In the case of a mistake, feedback is presented which steps were wrong only after six steps were completed. When no mistakes are made, a “Good” word is displayed, and the next trial is shown.

In the provided scripts, participants would practise the following two sequences: ←→↑↓→← and →↑←↑→↓. The script has been commented for easy alteration to include more sequences and/or sequences with more stimuli and responses. To counterbalance target stimuli positioning, both sequences are rotated four times, resulting in eight different counter-balanced sequences so that participants receive the same sequences but with different positions. Possible variations in sequence difficulty or foot strength/preferences are not expected to affect participants’ learning process. To give an example, the sequence ←→↑↓→← rotated once resulted in a different counter-balanced sequence ↑↓→←↓↑. Participants are given the liberty to organize their responses in the most naturalistic manner, using any combination of their two feet for correct execution.

The script is executed as a singular practice block which consists of 24 trials for each of the two sequences for a total of 48 trials with additional 4 NoGo trials. Halfway through each block (after 24 trials) the script would automatically execute a 30-second break before continuing to the second half. Both number of trials and break sessions/timing can be altered in the script. A completed block was indicated by a “This is the end of the session” message, followed by another feedback screen displaying the average response time and mistakes ( %) for the block after completion. Between training blocks, participants would be given a three-minute break, and after the fourth block, received a 10-minute break, in both cases controlled by the experimenter. Rest breaks are important to avoid physical and mental fatigue and participants were requested not to end breaks early. Experiments can have between 4 and 8 training blocks, which can depend on the amount of time that is allocated for training and the complexity of the sequence (longer sequence will take more time). A test phase which consists of 2 blocks of 48 trials can present sequences that are either familiar or unfamiliar, in which the blocks are counterbalanced between the participants. The test phase offers insight to the performances of learnt versus novel sequences.

If the main experimental goal is to focus on behavioral results, then the minimal setup with no further integration of hardware complexities is required. The details in previous sections should be sufficient to conduct the Dance-DSP task. However, if one is also interested in our MoBI setup, then the next sections can be read too.

### Wireless electroencephalography: ANT Neuro eego^TM^

In the last few years, mobile EEG technology reached a level of fidelity that accounts for movement artifacts and reliably allows for the investigation of cognitive mechanisms in naturalistic movement settings like dance [20]. We utilized a 22-channel ANT Neuro eego^TM^ sports EEG (ANT Neuro b.v., Hengelo, The Netherlands) amplifier and cap that was carried by the participant in a generic lightweight runner’s backpack with a Microsoft Surface Pro 2 tablet (See Fig. 2A). Should one's budget allow for it, more channels are possible. The tablet ran the ANT Neuro eego^TM^ mylab software recording data at 500 Hz (can be adjusted). The amplifiers had active shielding technology, which further increases the signal-to-noise ratio. The 22 channels utilized an adjusted 10–20 EEG montage. As we did not have dedicated electrooculogram channels, F9 and F10 were specified for eyeblinks. Channel CPz was used as the reference and channel FPz as the ground electrode in the current setup. As usual for EEG setups, impedances were kept below 10 kΩ.

### Markerless and wireless motion capture kinematics: Xsens MTw Awinda

The addition of motion capture can facilitate additional research goals aimed at kinematics like anticipatory predictors of balance control in motor sequence learning and performance. The integration of the Xsens MTw Awinda system is a natural extension with the Dance-DSP task for the understanding of limb kinematic synergies (for an extensive review of the Xsens technology see Paulich et al., [21] and Schepers et al., [22]). The Xsens MTw Awinda is a leading wearable, wireless inertial measurement system (IMS) that allows for three-dimensional (3-D) analysis of human movements [23]. The IMS system combines the use of multiple inertial sensors in the form of 3D-accelerometers, gyroscopes and magnetometers, that are attached to various bodily segments and provide accurate kinematical estimations. Combined with the MVN Analyze software (i.e., the data capture application), precise estimations of segment/ sensor displacement (x, y, z axes), velocity and angular acceleration can be acquired and extracted.

The current protocol is focused on capturing measurements of center of mass (CoM). We only outline the use of 7 sensors, which is the minimum required for accurate measures of lower body kinematics and CoM specified by Xsens MVN Analyze (Ver. 2021.0.1 build 6752). Should one require more sensors for investigating upper-body segments, up to 20 sensors can be used at once for a full-suit setup. When recording with 7 sensors, MVN Analyze has a maximum frame capture of 100 hertz. When using all 20 sensors the maximum output frame capture is 60 Hz [21]. The sensors used currently: center of the pelvis, left and right thighs, left and right shins, left and right foot. Data from the sensors are wirelessly transmitted through the Wi-Fi MTw Awinda base station – which represents one suit. A separate computer from the E-Prime^Ⓡ^ presentation is required to run the MVN Analyze software so that kinematical data can be recorded, processed and extracted for further analyses.

### Subjective self-reports: NASA Task Load Index and Affect Grid

We also captured subjective physical demand and effort via the commonly administered NASA-Task Load Index [24], and the Affect Grid [25] before and after every block to check participants’ arousal states and if they were overly fatigued from each training block. These self-reports provided us with an understanding of cognitive effort changes over time stemming from participation in the Dance-DSP task.

### Overall procedure

Participants were instructed to wash their hair with shampoo the day before or latest on the day of the experiment to help EEG data collection. All participants were briefed about the purpose of the study and were told their rights to leave at any time and then provided explicit written informed consent to experimentation. The usual EEG setup was completed with cap fitting, gelling, and a check for signal quality. Once the amplifier and laptop were placed into the runner’s backpack (Fig. 2A), they were asked to take their shoes off to prepare for motion capture calibration.

The 7 lower body sensors were then attached (Fig. 2A), and a calibration procedure for the Xsens was performed. The participant was instructed to stand straight, both arms hanging by the side (known as the N-pose) and wait for the researcher to start the procedure in MVN Analyze software. Once ready, the researcher signals to the participant to walk four meters in a straight line at normal walking speed to a predetermined marking on the ground, turn 180 degrees, and walk back to the starting position. MVN Analyze then processed the calibration procedure and notified when a good calibration result was achieved – otherwise the calibration procedure was repeated. The Dance-DSP task then starts the block with the monitor displaying the four rectangles and the participant learning the sequence (Fig. 2C).

Due to the nature of the LSL, E-Prime^Ⓡ^ was restarted after each block and the EEG recording was stopped and restarted to ensure an easy separation of blocks during pre-processing. This gave participants a 2–3-minute break in between blocks. Additionally, a 10-minute break was always offered halfway through the experiment for more rest and recovery.

### Video supporting the motivation, methods, and setup

Procedural video: https://www.youtube.com/watch?v=zFP1rWJ2FJ8

## Method validation

### Data latency checks

To report on data latency, the response onset delay between the initiation of responses on the dance-pad to when E-Prime^Ⓡ^ recorded the responses was on average 167.4 ms (± 20.8 ms; 2880 trials), in which E-Prime^Ⓡ^ accounts for as onset delays in its internal response time calculations. Because LSL was responsible for sending of event markers from E-Prime^Ⓡ^ to both MVN Analyze and EEG, we tested the time synchronization via sent/received timings on each respective recording device. The network delay for receiving event markers in and EEG after sent from E-Prime^Ⓡ^ was on average 7.3 ms (± 2.4 ms; 2880 trials), which falls within a normal range for local User Datagram Protocol (UDP) communications. Importantly, none of the participants reported any feeling of lag delays when responding to the stimuli.

### Pilot data validation checks

#### Participants

We captured data from 12 participants (6 female), average age 23.3 years (*SD* = 2.6), via convenience sampling from the University of Twente. Participants were healthy with no history of neurological, psychological or psychiatric disorders; no alcohol, tobacco, or other drug addictions or dependencies; no signs of cognitive impairment as well as no obvious physical injuries or impairments that would affect their performance. They should not have taken part in similar sequence learning studies. Participants were tested on their foot dominance by asking questions around ball-kicking and declaration of their lead leg if they skated or snowboarded and confirmed by having them perform a simple footedness test that involved first closing their eyes, standing straight and then pushing them from behind which typically caused them to take a step forward with their dominant foot [[26], [27]].

#### Behavioral exemplar results: response time and accuracy

Extraction of the data was merged for all participants to form a completed data frame. We show some exemplar results and visualizations at the group level, and factorial level analysis on learning blocks. The mean response times for whole sequence execution in the Dance-DSP task were relatively short – overall mean step-level response time for a sequence across all 4 learning blocks was 357.9 ms (*SD* = 182.9 ms), considering that mean key-press responses range between the 200 to 400 ms [[28], [29]].

**Fig. 3:**
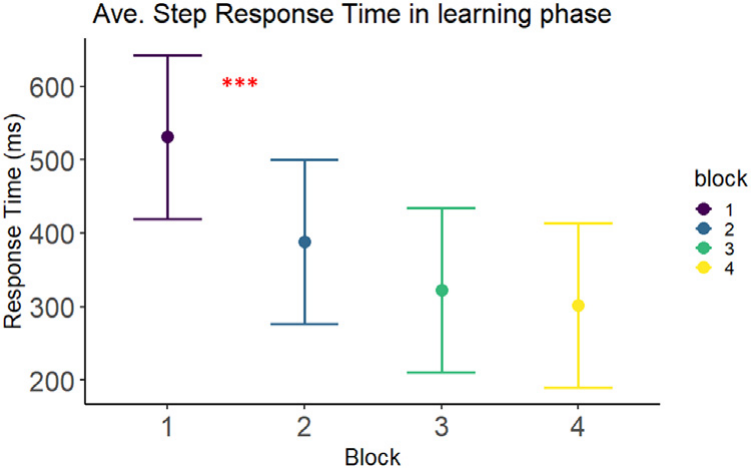
Visualization of a linear-mixed effects model of mean step-level response times across blocks for accurate sequence trials (all 6 steps) for 12 participants with 95 % confidence intervals.

**Fig. 4:**
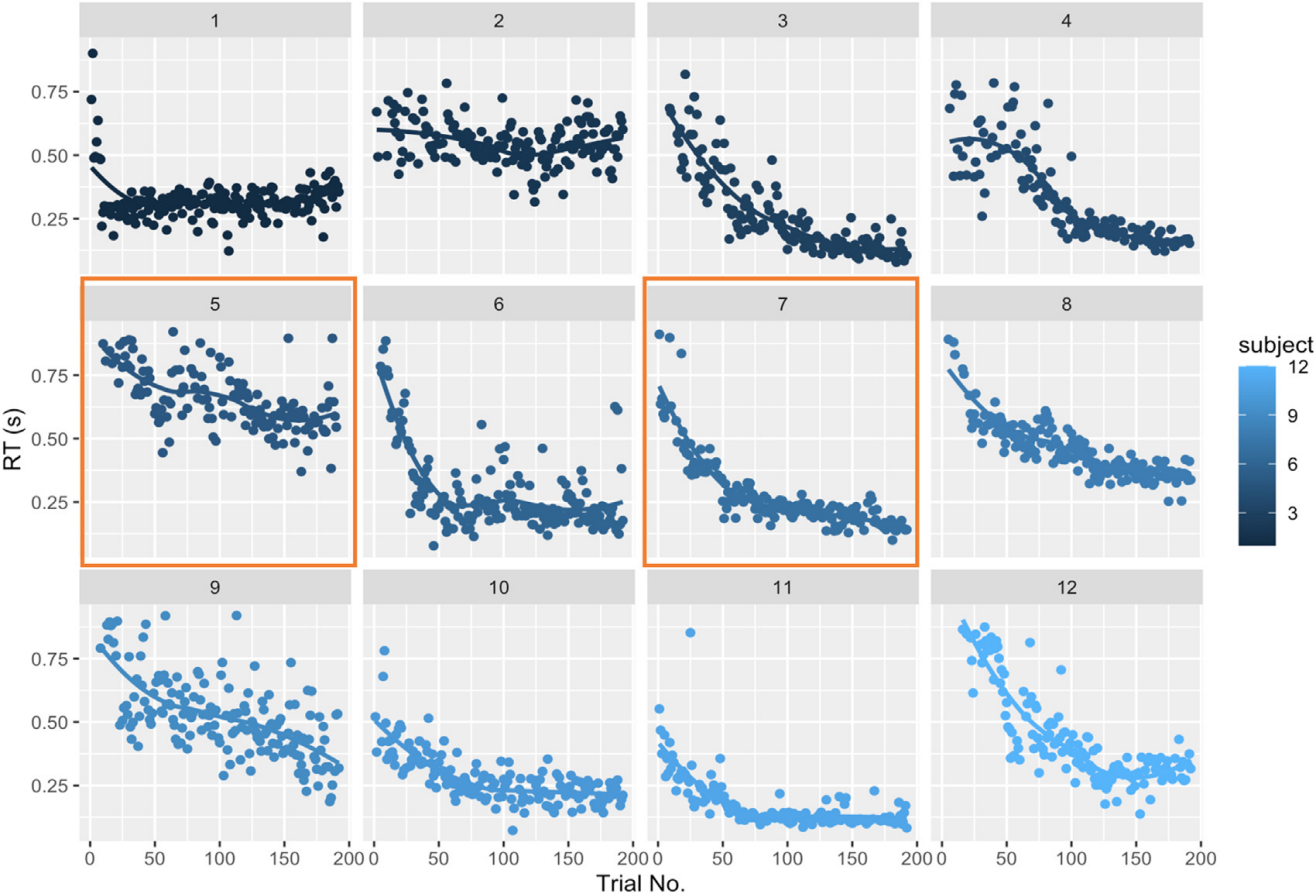
Visualization of response time (ms) for 192 trials (4 blocks x 48 trials) from the learning phase for each participant. For most participants, learning is inferred from the decrease in response times from the repetition of trials. Differences in response time changes between Participants 5 and 7 (orange boxes) suggest different learning styles or strategies/learning performance in the Dance-DSP task.

#### EEG exemplar results: theta event-related de/synchronization (ERD/S)

The EEG recordings were pre-processed using MATLAB Brainstorm version 24.03.2024. After importing the raw datafiles and channel positions, a bandpass filter of 0.3 – 30 Hz was applied. A new reference electrode was created based on the average of all electrodes. Next, an Independent Component Analysis (ICA) using the Infomax algorithm was applied. On average, 1.7 components were removed per participant and block. An epoch of 200 ms before the ‘Go’ cue marker serves as a baseline for the ERD/S analysis. A Morlet Wavelet time-frequency analysis was applied to all epochs, extracting the theta (4–8 Hz) frequency. Each epoch had a different length because we extracted exactly the time it took for participants to respond to all 6 steps. This approach was chosen over a fixed epoch length because behavioral data showed that sequence execution durations varied significantly, ranging from 500 ms to 6000 ms. To minimize edge effects in wavelet extractions, we extended the epoch duration by adding 100 ms of padding both before the first step and after the last step, ensuring a more stable time-frequency representation.

Following Yamauchi et al. [30], we utilized a method to perform Dynamic Time Warping (DTW) to standardize epoch lengths whilst retaining step level data. DTW is a technique which enables the alignment of two sequences holding different speeds or time lengths, making it possible to align the steps of two different sequences [31]. Classic DTW algorithms merely allow the comparison of two sequences at a time and furthermore result in the warping of datapoints, causing compression or stretching of data [30]. However, Yamauchi et al. [30] proposed reducing time series data to a fixed number of datapoints by averaging many small fragments within the sequence. In our study, the number of datapoints per epoch ranged from approximately 500 - 3000, based on epoch length ranging up to 6 s with a recording frequency of 500 Hz. We decided to reduce all epochs to 300 datapoints because our epochs were longer than those in the referenced study, which used 100 and 200 datapoints. This led to a process of averaging an entire sequence into 300 datapoints. The process involves placing 300 equally spaced ‘bins’ throughout all datapoints and calculating the average within each bin, resulting in all epochs containing 300 datapoints of the theta frequency power. Subsequently, the 300 datapoints formed six larger bins to obtain an average theta frequency estimate per step.

After applying DTW to each epoch, the final step was to calculate the ERD/S percentage with reference to the baseline of −200 ms before the ‘Go’ cue. The following formula was used, with Power_timepoint_ representing the raw theta frequency average per step, previously calculated:

$$\text{ERD}/S\left(\%\right)=\left({\text{Power}}_{\text{timepoint}}-{\text{Power}}_{\text{baseline}}\right)/{\text{Power}}_{\text{baseline}}\times 100$$

**Fig. 5:**
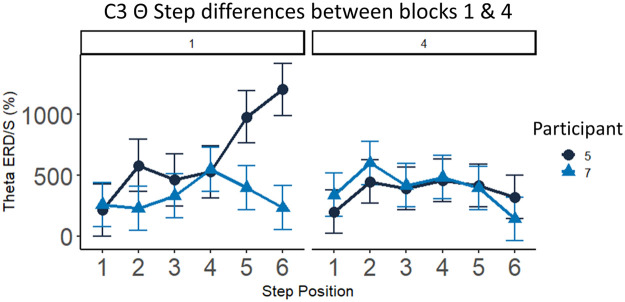
Average theta de/synchronization (%) for each dance-step position over C3 electrode, comparison between participants 5 and 7. The figures showcase divergences in the theta activation in the 1st block for Steps 5 and 6. In the 4th block, theta power was largely the same between both participants.

This resulted in a dataset containing the theta ERD/S percentage per step for every participant and block for each electrode. In the Fig. 5 below, we showcase a comparison of theta ERD/S differences at the C3 electrode between Participants 5 and 7 for each step.

#### Kinematics exemplar results: sequence-level center of mass velocity changes with training

**Fig. 6:**
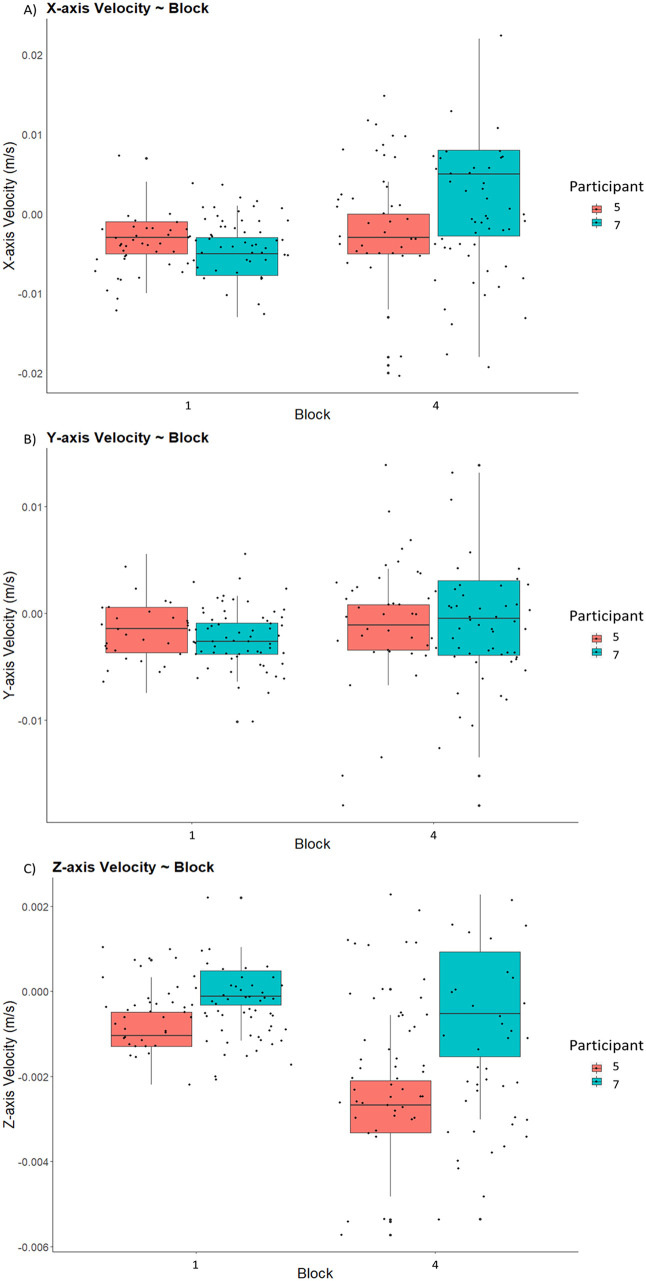
Average sequence center of mass velocity (m/s) in the A) x-axis, B) y-axis and C) z-axis for a comparison between participants 5 and 7. This data was obtained from the Xsens MTw Awinda motion capture system. This is a follow-up comparison of earlier learning curves in Fig. 4. The figures show different patterns, in that participant 7 shows development of more variability in the velocity of x, y and z-axes compared to participant 5 from block 1 to block 4. The changes between block 1 and 4 for participant 7 could be linked to Bernstein’s classical theories of freeing of degrees of freedom with expertise development [33,34].

Xsens data was extracted using the MVN Analyze software package, utilizing its built-in export function to obtain displacement, velocity, and acceleration variables. Since we recorded each block performance as an individual file – all 48 trials were captured, and with the help of event markers, clear segregation of individual dance-step/ sequence level performance is possible. In Fig. 6 below, we visualize CoM velocity between exemplar Participants 5 and 7 due to their earlier behavioral differences for sequence level execution for training blocks 1 and 4 to highlight individual learning differences in kinematics.

## Limitations

The goal of this manuscript is to facilitate researchers that are interested in motor sequence learning of the implementation of a Dance-DSP task. We aimed to transfer the same task properties and general motor learning phenomenon into a more naturalistic format beyond keyboard-based laboratory settings and provided detailed methodology here. Importantly, our methodology contributes to the burgeoning trend of porting other motor sequence learning tasks that have been shown to be feasible [16] and learning differences between different populations [14]. The Dance-DSP task, together with the MoBI application, seems a very promising approach for the investigation of motor sequence learning and the investigation of the synchronies between body and mind. We hope more researchers will utilize such paradigms and/or be inspired to adapt existing ones to further motor neuroscience research.

The first limitation of using the Dance-DSP task is the physical effort and length of time for the experiment. In the past, key press tasks focused on the ability to mainly utilize cognition and mental effort to perform the sequence learning task. In the Dance-DSP, there is a need to plan for the physical capacities of the individual to perform the standing/ whole-body movement. Olivier et al., [16] showed that in an experiment consisting of 6 blocks on a single day of practice with 864 steps was tolerable and feasible for their participants. In the current Dance-DSP task, we had 4 training blocks and 2 test blocks in a session with 1728 steps in total – double the steps from Olivier et al., [16]. The whole procedure including participant setup took approximately *2.5 – 3 h* in total. We consider *this as the recommended upper limit of tolerance* for younger participants for continual focus on performing the Dance-DSP task. Participants reported that general tiredness progressed during the experiment, but no adverse events were reported (from the NASA-TLX). We do not recommend sessions to be longer than 3 h in total. From the exemplar results in Fig. 3, we also estimate that general learning plateaued with 4 learning blocks, which indicates the trials were adequate to elicit most of the learning. We also followed a rule of giving participants approximately three-minute breaks between blocks, aiming to account for about >85 % of recovery based on normal activity energetic systems utilization and recovery [32]. Halfway through the experiment, we administered a mandatory longer break lasting 10 min and offered a sugary drink for recovery of glucose stores.

A second limitation would be the EEG caps and amplifiers used, as there was still a need to be conscientious about giving instructions regarding excessive head movements. In our pilot testing, we noticed participants with rapid and large head movements inducing unwanted artifacts in the EEG data. Thus, we improved the instructions and told participants to keep their head still and focus on the lower body in executing the Dance-DSP task. With this, participants became more aware to minimize their head movements and not generate large unwanted artifacts. In the future, we think that EEG technology and algorithms would advance to a level that would account for large overt actions and automatically correct these artifacts in real-time with precision [35].

One of the potential use cases of the naturalistic Dance-DSP task is to make training more interactive in diverse populations like the elderly, individuals with Parkinson’s or stroke. It may reap additional clinical benefits like improved dynamic balance and control. For example, Granacher et al., [36] showed that participation in 8-weeks of progressive salsa dancing program enhanced both static and dynamic postural control in the elderly. In general, the efficacy in dance or dance-like programs are positive and suggest that participation in these kinds of activity can lead to better management of dynamic mobility, overall physical performance and balance improvements [37]. It is therefore possible that the Dance-DSP task, in an applied sense, can act as an adjunctive dynamic mobility motor learning program (with progressive training) that may lead to improvement of balance control and a reduction of falls risk. However, the efficacy, training volume, intensity etc. have yet to be tested adequately in these diverse populations and one must err on the side of caution. Physiologists and ergonomists may provide an interdisciplinary view on the exertion and rest ratios for recovery and optimal learning performance in these diverse populations when using the Dance-DSP task.

## Ethics statements

All participants provided explicit written informed consent to collect physiological and behavioral data, were briefed about the purpose of the study, and were told their rights to leave at any time during the study. All procedures were performed in accordance with Human Ethics guidelines approved by the University of Twente Ethics Committee filed under No. 210,390.

## Declaration of generative AI and AI-assisted technologies in the writing process

During the preparation of this work the author(s) used Microsoft’s Copilot for textual content primarily to refine paragraph structure and organise thoughts without influencing intellectual direction of the manuscript. This approach underscores a judicious application of AI tools to augment the research process, maintaining the integrity and originality of the work. After using this tool/service, the author(s) reviewed and edited the content as needed and take(s) full responsibility for the content of the publication.

## CRediT authorship contribution statement

**Russell W. Chan:** Conceptualization, Methodology, Software, Supervision, Funding acquisition, Writing – review & editing. **Victoria Lakomski:** Investigation, Validation, Methodology, Formal analysis. **Johannes V.R. Pannermayr:** Investigation, Validation, Methodology, Formal analysis. **Emma Wiechmann:** Investigation, Validation, Methodology. **Jan-Willem J.R. van ‘t Klooster:** Methodology, Writing – review & editing. **Willem B. Verwey:** Conceptualization, Writing – review & editing.

## Declaration of competing interest

The authors declare that they have no known competing financial interests or personal relationships that could have appeared to influence the work reported in this paper.

## Data Availability

Data will be made available on request.

## Appendix Supplementary materials

Image, application 1
