# Supplementary material for: Multimodal mobile brain and body imaging for quantification of dance motor sequence learning
Source: MethodsX. 2025 Apr 19;14:103324. doi: 10.1016/j.mex.2025.103324 (PMC12053982; doi:10.1016/j.mex.2025.103324)
Supplement: Supplementary file 1 [file mmc1.docx]

**Appendix 1: Equipment list**

Basic setup

| **Equipment name** | **Make** | **Model** | **Comment** |
| --- | --- | --- | --- |
| E-Prime® | Psychology Software Tools | Minimum Ver. 2.0 (3.0 preferred) | For creating the stimuli executing the experiment |
| Display for stimuli | LG (in this experiment) | Model nr. OLED77CX6LA (77 inch display) | Any computer monitor/ TV that has at least 60Hz refresh rate |
| Dance mat USB device | D-Force | Nonslip Deluxe Dance Pad Ver. 5 (<https://dancepadmania.com/deluxe/>) | Multiple options from retailers but choose one that is non-slip and of higher quality |
| USB Input mapper | JoyToKey | Ver. 5.2.1 | PC software used to map the dance mat as keyboard input |
| Personal computer (PC) | Any brand | Any model | Ensure PC has the minimum Windows OS requirement to run E-Prime® 2/3 |
| Weighting scale and tape measure for height | Any brand | Any model | To record individual differences |

Optional complexities:

| **Equipment name** | **Make** | **Model** | **Comment** |
| --- | --- | --- | --- |
| EEG Amplifier | ANT Neuro | eego^TM^ sports EEG | This amplifier supports Mobile Brain/ Body Imaging due to its battery pack that lasts abour 3-4 hours. |
| EEG Recording software | ANT Neuro | mylab | This is the standard recording software supplied with ANT Neuro’s amplifier and has LSL protocols for receiving markers from stimuli software. |
| EEG Recording Tablet | Microsoft | Surface Pro 2 | Supplied with ANT Neuro’s eego^TM^ amplifier |
| 3D Motion Capture | Movella Xsens | MTw Awinda system (up to 20 sensors) | The motion capture system gives access to kinematics of limb positioning such as velocity, acceleration and displacement |
| 3D Motion capture software | Movella Xsens | MVN Analyze (on a yearly subscription) | The software allows for capture of 3D recordings, analysis and export of data files. |
| Wi-FI Ethernet router network hub | Any major brand e.g. Linksys, TP-Link etc. | Any model that has at least 4 ethernet slots (and an ethernet cable) | The hub is for communications between the motion capture computer and the stimuli computer for sending and receiving event markers |
| Personal computer for motion capture recording (PC) | Any major brand | Ensure that the computer has the latest Windows 10/11 OS | This computer should have enough processing power (≥ Intel i7 in 2021), ram (≥16gb) and enough storage for motion capture |
